# Organization of Prenatal Care in Orofacial Clefts and Suspected Robin Sequence: A European Survey

**DOI:** 10.1097/SCS.0000000000011312

**Published:** 2025-03-27

**Authors:** Shirley van de Velde, Christine L. van Velzen, Peter G. Scheffer, Aebele B. Mink van der Molen, ERN CRANIO Working Group on Clefts

**Affiliations:** * Department of Pediatric Plastic and Reconstructive Surgery, University Medical Center Utrecht, Wilhelmina Children’s Hospital; † Department of Obstetrics and Gynecology, University Medical Center Utrecht, Wilhelmina Children’s Hospital, Utrecht, The Netherlands

**Keywords:** Europe, orofacial clefts, prenatal diagnosis, robin sequence, survey

## Abstract

Advances in prenatal imaging and genetic testing have improved the detection of orofacial anomalies, allowing for early diagnosis and comprehensive counseling. This study aims to provide an overview of current prenatal care practices for orofacial clefts and/or suspected Robin sequence (RS) across European countries and to identify disparities to inform future improvements. A cross-sectional survey was distributed to health care professionals affiliated with the European Reference Network CRANIO, collecting data on prenatal imaging, genetic testing, counseling, and termination of pregnancy. Responses were obtained from 31 health care professionals in 27 hospitals across 17 European countries. All participating countries have some sort of prenatal screening program. Ultrasound examination was performed at 1 or 2 timepoints during pregnancy, with varying techniques used to assess orofacial structures. Fetal MRI was performed in 59% of centers, primarily for multiple (severe) anomalies. Centers utilizing specific imaging techniques reported fewer missed diagnoses. Prenatal genetic testing was available in 96% of centers, with array-based copy number variation and whole-exome sequencing performed in 59% and 52% of centers, respectively. Nearly half of the centers provided multidisciplinary counseling, with team composition and session frequency varying widely. Pregnancy termination for orofacial clefts or micro-/retrognathia was not legal in 7 countries. Termination rates and trends differed across centers. In conclusion, variability exists in prenatal care for orofacial clefts and/or suspected RS across Europe, particularly in imaging techniques, genetic testing, and pregnancy termination practices. These findings highlight the need for standardized guidelines and equitable access to multidisciplinary care to optimize outcomes for affected pregnancies.

Orofacial clefts are among the most common congenital anomalies, affecting ~1 in 700 live births worldwide.^1^ These anomalies can lead to significant challenges, such as feeding difficulties, speech and hearing impairments, and psychosocial issues.^2^ In certain cases, orofacial clefts are associated with additional congenital anomalies or syndromes, including Robin sequence (RS), which may complicate postnatal management. RS is characterized by a triad of micrognathia, glossoptosis, and airway obstruction, often occurring alongside cleft palate.^3,4^ Infants with RS require specialized care due to airway and feeding difficulties.

Advances in prenatal imaging, particularly detailed ultrasound and fetal MRI, have improved our ability to detect orofacial clefts and signs of micrognathia or retrognathia during pregnancy.^5–8^ In addition, other structural anomalies are better visualized. Parallel to these imaging advancements, developments in prenatal genetic testing techniques have expanded our knowledge of genetic variants associated with these anomalies, enabling detailed prenatal diagnoses.^9^ Consequently, parents can now receive comprehensive information early in pregnancy about the presence of orofacial clefts and suspected RS, as well as associated anomalies or genetic variations as part of a syndromic diagnosis. This early insight allows parents and health care professionals to make more informed decisions about whether to continue the pregnancy and to better prepare for the course of pregnancy, delivery, and postnatal care.

Because of technical innovations, prenatal screening and diagnostics have advanced significantly over the last 2 decades, yet there is no clear overview of how prenatal care for orofacial clefts and suspected RS is organized across European countries. Given the complexity of prenatal decision-making in cases involving orofacial clefts and/or suspected RS, the ERN CRANIO (European Reference Network for rare and/or complex craniofacial anomalies and ear, nose, and throat disorders) scientific committee has recognized the need for a comprehensive understanding of how prenatal care is organized across Europe.^10^

The aim of this survey study is to provide an overview of the current organization of prenatal care for pregnancies diagnosed with an orofacial cleft or suspected RS across European countries. This study will also serve as a foundation for future discussions on existing disparities in prenatal care, ultimately guiding efforts to optimize prenatal care practices and create more uniform care.

## MATERIALS AND METHODS

This cross-sectional study utilized an online survey developed by the author panel and further refined by local clinicians and the scientific committee of the ERN CRANIO. The final survey contained a mixture of a maximum of 59 multiple-choice and open-ended questions, tailored based on respondents’ answers (Supplement 1, Supplemental Digital Content 1, http://links.lww.com/SCS/H609). Most questions were structured to prompt respondents to provide information about practices at their clinic, assuming standardized definitions and management within the clinic. The survey was created and distributed using Microsoft Forms, an online survey platform. The survey was available only in English.

The survey was sent to member hospitals and supporting partners within the ERN CRANIO cleft workstream (i.e., those involved in the care of infants with clefts and suspected Robin sequence). The network encompasses professionals such as clinical geneticists, gynecologists, nurse specialists, oral and maxillofacial surgeons, otolaryngologists, pediatricians, (pediatric) plastic surgeons, and psychologists. Initial invitations were sent on October 11, 2024. Responses were collected anonymously. A reminder was sent on October 28, 2024 to increase response rate. During the ERN CRANIO 9^th^ annual meeting in Gdansk, Poland (November 6–8, 2024), a link to the survey was distributed to all participants.

Both complete and incomplete responses were included in the analysis, except for those deemed unreliable by the authors (e.g., only respondent characteristics were completed or <50% of the survey was filled out). Multiple responses from one center were allowed, given the fact that for example an oral and maxillofacial surgeon may not be able to answer all questions regarding prenatal genetic testing.

Approval for this study was obtained from the UMC Utrecht (ethics code: 24U-1905). This study does not fall under the scope of the Dutch Medical Research Involving Human Subjects Act (WMO). It, therefore, does not require approval from an accredited medical ethics committee in the Netherlands. However, in the UMC Utrecht, an independent quality check has been carried out to ensure compliance with legislation and regulations. All participants consented that their responses could be used for research purposes.

## RESULTS

### Respondents

**FIGURE 1:**
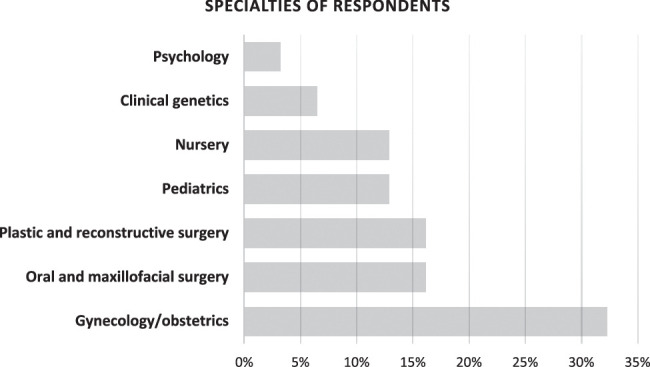
Distribution of specialties of respondents.

A total of 31 health care professionals from 27 ERN member/partner hospitals spread across 17 European countries completed the survey, with no exclusions made. An overview of the respondents’ characteristics can be found in Supplemental Table 1 (Supplemental Digital Content 2, http://links.lww.com/SCS/H610). Most respondents originated from the Netherlands (n=5, 16%) and Spain (n=4, 13%). The majority of the respondents were specialists in the field of gynecology/obstetrics (32%), oral and maxillofacial surgery (16%), or plastic surgery (16%) (Fig. 1). Other fields included genetics (7%), nurse specialists (13%), pediatrics (13%), and psychology (3%). All except 2 respondents, who worked in a general hospital and a specialized pediatric hospital, were affiliated with university hospitals. In nearly half of the centers (n=12, 44%), a specialized multidisciplinary team was available. The incidence of prenatally detected orofacial clefts and/or micro-/retrognathia varied significantly, ranging from 1 to 2 cases per year to more than 21 cases annually (Supplemental Table 1, Supplemental Digital Content 2, http://links.lww.com/SCS/H610).

### Prenatal Screening Programs

All countries in this survey reported the existence of some form of prenatal screening program. Programs were introduced as early as the 1970s or as late as the 2000s. In 53% of the countries, prenatal screening is fully covered by social health insurance. In Austria, the costs are covered by private or additional health insurance, or out-of-pocket payment. Ultrasound examinations are carried out in Austria at cost price and paid for out-of-pocket. In the United Kingdom, the costs are covered by the National Health Services (NHS). In Hungary and Poland, only essential ultrasound examinations are typically covered; more advanced imaging or genetic screening, such as noninvasive prenatal testing (NIPT), fetal MRI, or in-depth genetic testing, may require private or additional health insurance or out-of-pocket payment. In Sweden, there is regional variability, particularly in the maternal age threshold for trisomy screening coverage. In many countries (n=13), prenatal care is provided both in centralized centers (i.e., university hospitals) and in dedicated clinics (i.e., clinics offering prenatal care only). For example, in France there is a prenatal diagnostic center for pregnancies with pathology. In this dedicated center, multidisciplinary care is provided. In other countries, such as Latvia, prenatal screening is performed in dedicated clinics; however, high-risk patients are referred to university hospitals. Respondents did not report if there was a difference in reimbursement for prenatal care offered in centralized centers or in dedicated clinics.

### Imaging

In 71% of the countries (n=12), a prenatal screening ultrasound is routinely performed at a single timepoint (15–16 wk or 18–22 wk gestation). In 29% of the countries (Germany, Hungary, Latvia, the Netherlands, and Poland), 2 screening ultrasounds are routinely performed (11–14 wk and 18–22 wk gestation). In case of abnormalities, diagnostic ultrasound is frequently performed by gynecologists/obstetricians (78% of the centers) and sonographers (33% of the centers) (Supplemental Table 2, Supplemental Digital Content 2, http://links.lww.com/SCS/H610). Several centers reported techniques for evaluating the palate (Supplemental Table 3, Supplemental Digital Content 2, http://links.lww.com/SCS/H610), including the “the equal sign”, “Batman sign”, “search the mouth bell”, and specific sagittal or axial transverse views. For mandible assessment, the maxilla-nasion-mandible (MNM) angle was reported frequently. Other techniques reported include the inferior facial angle (IFA), “jaw index”, and “mandible length”. One center published these techniques.^11^

Fetal MRI, performed on indication in 16 centers (59%), is typically conducted between 28 and 32 weeks gestation. Common indications include severe micrognathia, microsomia, suspected central nervous system anomalies, airway-compressing masses, or syndromic conditions.

**FIGURE 2:**
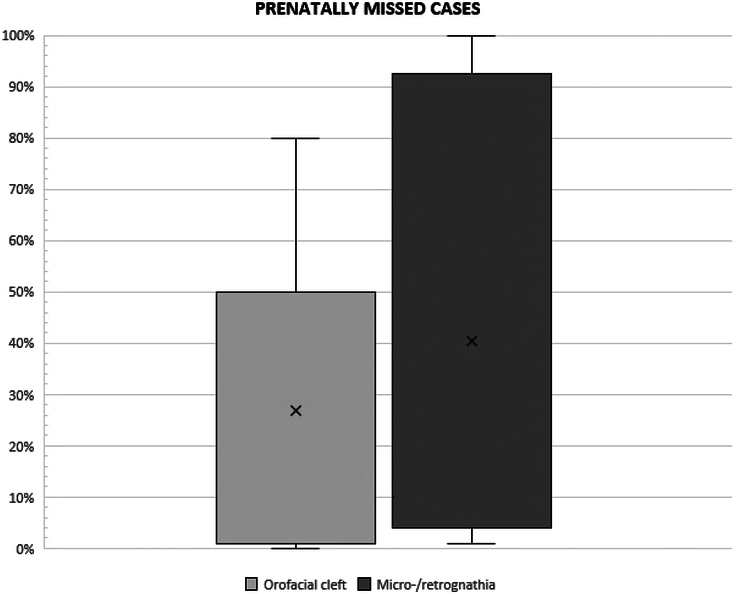
The range of prenatally missed cases (orofacial cleft versus micro-/retrognathia).

Figure 2 illustrates the range of prenatally missed orofacial cleft and micro-/retrognathia cases (estimated number). The estimated number of prenatally missed cases of orofacial cleft and micro-/retrognathia per center can be found in Supplemental Table 3 (Supplemental Digital Content 2, http://links.lww.com/SCS/H610). Centers with lower rates of prenatally missed cases reported more frequent use of specific techniques for assessing the palate and mandible.

### Genetic Testing

Prenatal genetic testing is available in 96% of the centers (n=26), with clinical geneticists or gynecologists (or both) conducting counseling for genetic testing in 96% of the centers (Table 2, Supplemental Digital Content 2, http://links.lww.com/SCS/H610). Testing is almost always (71%–100%) recommended for orofacial clefts in several centers, particularly in Belgium, France, Hungary, Ireland, Latvia, the Netherlands, Slovenia, and Spain. Respondents indicated that genetic testing was offered less frequently in cases of micro-/retrognathia, except for one center in France. This center reported offering genetic testing “very frequently” for clefts (51%–70% of cases), and offering genetic testing “almost always” for micro-/retrognathia (71%–100% of cases). Factors as cleft severity, additional anomalies, family history, and findings on diagnostic imaging play an important role in decision-making. Maternal age seems to play a limited role (i.e., for 32%–35% of the respondents, it is a decisive factor). In general, parents frequently or almost always opt for genetic testing for orofacial clefts or micro-/retrognathia (n=20 versus n=17, respectively). In Austria, Norway, and Poland parents opt for genetic testing less frequently (10%–30%).

Advanced techniques such as array-based copy number variation (CNV) and (trio-) whole-exome sequencing (WES) are performed in 59% and 52% of the centers that perform genetic testing, respectively (Supplemental Table 2, Supplemental Digital Content 2, http://links.lww.com/SCS/H610). Relevant findings lead to additional counseling sessions in half of the respondents’ centers, focusing on perinatal care planning and termination options (both in 67% of the centers) or additional imaging such as detailed anomaly ultrasounds or MRI (30%). Notably, most respondents (n=25, 81%) reported the absence of national guidelines for genetic testing in prenatal orofacial clefts or micro-/retrognathia diagnoses.

### Counseling

In nearly half (44%) of the respondents’ centers, prenatal counseling is provided by a multidisciplinary team comprising specialists such as oral and maxillofacial surgeons, clinical geneticists, plastic surgeons, gynecologists/obstetricians, pediatricians, nurse specialists, and speech-language pathologists. Counseling frequency varies, with sessions offered once (13%), 2 to 3 times (35%), or on an as-needed basis (35%). Three centers (Austria, Finland, and Latvia) lack follow-up procedures to ensure parental understanding. Adequate interdisciplinary support was reported by 74% of respondents, especially in centers with multidisciplinary teams, although opinions varied within one such center.

### Termination of Pregnancy

In 7 countries (Belgium, Finland, Ireland, Poland, Portugal, Slovenia, and the United Kingdom), termination of pregnancy (TOP) for orofacial clefts or micro-/retrognathia is not permitted according to the survey respondents. France allows TOP for micro-/retrognathia only. Legal time limits for TOP ranged from 18 to 24 weeks, with Sweden having the strictest limit (18 wk) and France the most permissive (42 wk). TOP for these diagnoses was rare, occurring in 0% to 30% of cases. Trends in TOP vary across centers, with some centers (in the Netherlands, Slovenia, Spain, and Sweden) reporting an increase over time, whereas others (in Hungary, Norway, and Spain) observed a decline.

## DISCUSSION

This cross-sectional survey study was designed to provide a comprehensive overview of the organization of prenatal care for pregnancies diagnosed with orofacial clefts and/or micro-/retrognathia across European countries. Although all surveyed countries have some form of prenatal screening program, including voluntary noninvasive prenatal testing (NIPT) and standard fetal anatomy ultrasound examination(s), the findings reveal notable variability in screening performance of imaging, genetic testing, and counseling practices, alongside marked disparities in termination of pregnancy regulations.^12–16^ This could be caused by differences in procedure performance and coverage of health care costs. An in-depth analysis of the regulations per country was beyond the scope of this survey, but such an effort would certainly provide important additional information and might serve as a foundation for a new European guideline to reduce practice variation between countries.

As expected, the number of prenatally detected orofacial clefts and micro-/retrognathia per year differed between the expertise centers. Therefore making it questionable whether smaller-sized centers have the same experience in prenatal care as larger-sized centers. Yet, when looking at the availability of standardized local institutional guidelines for prenatal care, this is lacking in both the smaller-sized centers as the larger-sized centers. In addition, the number of prenatally missed cases did not seem to differ between smaller-sized and larger-sized centers, according to this survey.

The results in this study revealed marked variations in the estimated rate of prenatally missed cases of orofacial clefts and/or micro-/retrognathia, despite 2D or 3D ultrasound and fetal MRI being commonly used diagnostic tools. These findings suggest that detection rates vary between centers, which may be caused by many factors not assessed in depth in this survey. One possible explanation for this variability is that some respondents may have misinterpreted the survey question regarding the number of prenatally missed cases. Specifically, it seems that some participants reported the number of cases detected prenatally (e.g., 95% for cleft lip, 5% for isolated cleft palate) rather than the number of missed cases. In contrast, the variability observed in this survey aligns with findings in the literature, which indicate international differences in prenatal detection rates for orofacial clefts. For instance, the detection rate for CL±P has been reported as 71.7% in Denmark, 89.5% in England, and 44.6% in Switzerland.^8,17,18^

It seems that centers using specific ultrasound techniques for assessing the palate and mandible reported fewer missed cases, suggesting that advanced imaging protocols improve diagnostic accuracy. For example, techniques such as the “equal sign” or axial transverse ultrasound view for palate assessment and the MNM angle or IFA for mandible evaluation seem to provide critical diagnostic information.^11,19–21^ It remains unclear why centers have not adopted these methods and criteria more widely. However, discrepancies persist even among centers using similar imaging modalities, indicating that factors such as performer-experience, equipment quality, and training protocols may also play a role. Notably, in current literature, it seems that larger sample sizes and, therefore, more experience lead to a significant improvement in the test’s predictive accuracy. In a study with sample sizes involving more than 3000 patients, the predictive accuracy of the ultrasound ranged from 63% to 75%. In contrast, a study with 141 patients has shown a prediction accuracy of only 31%.^22^ Furthermore, fetal MRI is often considered a complementary tool for diagnosing orofacial clefts, particularly cleft palate, in fetuses at risk.^23–25^ Although some of the participating centers in this survey reported utilizing fetal MRI, this study did not assess its additional value or its impact on the number of prenatally missed cases in detail. Consequently, no conclusions can be drawn on its role in improving diagnostic accuracy. In addition, it is likely that cases of orofacial cleft or micro/retrognathia missed on initial ultrasound are not referred to tertiary centers for further evaluation with detailed ultrasound or fetal MRI. Moreover, cost and resource constraints are also likely contributors to the marked variations in detection rates, particularly in countries where advanced imaging is not covered by social health insurance. For example, in Hungary and Poland, only essential ultrasounds are typically funded, and parents may bear the financial burden of additional imaging.

The widespread availability of prenatal genetic testing, with whole-exome sequencing (WES) and array-based copy number variation (CNV) analysis commonly used, underscores the advanced capabilities of many centers in this field. Although these tests identified genetic variants, they did not significantly alter pregnancy management or treatment decisions in this study. The high rate of parental agreement to testing suggests that families value genetic information, possibly for postnatal planning or future family planning decisions. However, lower agreement rates in certain countries may reflect cultural, socioeconomic, or educational disparities. Besides, the majority reported that their country lacks a national guideline on performing (prenatal) genetic testing in cases with orofacial clefts or micro-/retrognathia. Yet, there are European guidelines available, through the ERN CRANIO, for both orofacial clefts and Robin sequence. These guidelines recommend that all cases should be referred to a clinical geneticist for counseling on prenatal genetic testing (especially with new techniques being developed and working towards the availability of WGS).^26,27^

Termination of pregnancy (TOP) for orofacial clefts or micro-/retrognathia was not permitted in 7 European countries according to the respondents in this survey. Yet, these findings do not align with current regulations in some of these countries.^28–32^ In many countries, voluntary termination is permitted up to a certain gestational age, and afterwards it is only allowed for specific (medical) indications. It is likely that conditions such as orofacial cleft and micro- or retrognathia are not considered valid medical indications in these contexts. The prohibition of termination of pregnancy in certain countries may reflect underlying religious or cultural influences. Although this study did not specifically investigate the role of religion, it is plausible that religious or sociopolitical factors influence these regulations. The variability in legal limits for TOP, ranging from 18 weeks (Sweden) to 42 weeks (France), also suggests significant ethical and cultural differences across countries. Further studies are needed to explore these influences on the availability of TOP and parental decision-making, providing context for national and cross-border differences in prenatal care options. Not only legal and religious issues play an important role in the decision to terminate a pregnancy but also the parental attitude towards orofacial clefts or micro-/retrognathia.^33^ This view is influenced by family, friends, and medical counseling provided by a health care professional.^34^ According to previous literature, parents who opted for a follow-up counseling session were less likely to terminate.^35^ In this present study, this was not examined.

Counseling practices varied widely. More than half of the centers lacked a formal protocol, which raises concerns about the consistency and quality of information provided to parents. This inconsistency could potentially influence parental decisions, including whether to continue the pregnancy. Moreover, differences in the frequency of counseling sessions may lead to varying levels of parental understanding and preparedness, which could have long-term implications for both families and health care systems. Furthermore, a multidisciplinary cleft or craniofacial team was present in only 44% of the centers. Counseling by multidisciplinary teams is a strength, as it ensures comprehensive input from various specialists. This is supported by our findings, as adequate interdisciplinary support was more often reported by centers with multidisciplinary teams. Yet, based on this survey no differences in the number of terminated pregnancies were seen between the participating centers with and without a protocol or multidisciplinary cleft or craniofacial team, but differences may exist when investigating this issue more in depth. In addition, previous studies reported that of those who had a prenatal diagnosis and counseling, up to 96% felt well prepared and/or considered prenatal diagnosis a benefit, especially when counseled by a multidisciplinary team.^36–38^

Several limitations of this study have to be addressed. First, the sample size was small due to the decision to distribute the survey exclusively to member hospitals and supporting partners from the ERN CRANIO cleft workstream. Although the aim was to gather responses from all European countries affiliated, this was not achieved despite sending reminders and distributing the survey during an annual meeting. As a result, the sample may not be fully representative. In addition, in some cases, only a single center from a given country completed the survey. One could question the extent to which these results are generalizable to national practice, as variation likely exists between centers within the same country. Moreover, it was not explicitly mentioned whether respondents should answer individually or on behalf of their entire cleft or craniofacial team. As a consequence, multiple responses were obtained from 3 centers, which resulted in discrepancies in answers to some questions. Similarly, multiple centers from the same country participated, leading to inconsistencies in responses to questions about national regulations or guidelines. In addition, some questions may—in retrospect—not have been formulated clearly enough. For instance, it seemed unclear for some respondents that we wanted to know how many cases with an orofacial cleft or micro-/retrognathia were missed prenatally. Consequently, some respondents reported the number of cases detected prenatally (e.g., 95% for cleft lip, 5% for isolated cleft palate) instead of the missed cases. Furthermore, the survey was available only in English, which may have affected how some questions were interpreted. Lastly, the reliance on self-reported data may introduce bias, as respondents might overestimate the quality of care provided in their centers. The strengths of this survey study are that it is the first questionnaire gaining insight in the current practices in prenatal care in Europe, and its broad geographic scope, which ensures a comprehensive overview of these current practices in different regions.

## CONCLUSION

Despite several limitations, the results confirm that substantial disparities in the organization of prenatal cleft and craniofacial care remain across Europe. This study underscores the need for harmonized prenatal care practices across Europe, with a focus on improving screening and diagnostic accuracy, and standardizing counseling protocols. Up-to-date (inter)national clinical practice guidelines on this subject are lacking and a multidisciplinary cleft or craniofacial team for counseling is not available in some of the participating reference centers. Addressing these gaps requires a coordinated effort among clinicians, researchers, and policymakers to ensure equitable, high-quality care for affected pregnancies. Standardizing practices and fostering partnerships are essential steps toward a more unified European approach to prenatal care. Future efforts should include consensus meetings to align and improve the organization of prenatal care across Europe.

## Supplementary Material

SUPPLEMENTARY MATERIAL

## References

[1] Mossey PA . Global perspectives in orofacial cleft management and research. Br Dent J 2023;234:953–957 37349453 10.1038/s41415-023-5993-4

[2] Agbenorku P . Orofacial clefts: a worldwide review of the problem. ISRN Plast Surg 2013;1–7

[3] Robin P . La chute de la base de la langue consideree comme une nouvelle cause de gans la respiration naso-pharyngienne. Bulletin de L’Académie Nationale de Médecin 1923;89:37–41

[4] Poets CF Wiechers C Koos B . Pierre Robin and breathing: what to do and when? Pediatr Pulmonol 2021;57:1887–1896 33580741 10.1002/ppul.25317

[5] Descamps MJ Golding SJ Sibley J . MRI for definitive in utero diagnosis of cleft palate: a useful adjunct to antenatal care? Cleft Palate Craniofac J 2010;47:578–585 20509765 10.1597/09-070

[6] Wang G Shan R Zhao L . Fetal cleft lip with and without cleft palate: comparison between MR imaging and US for prenatal diagnosis. Eur J Radiol 2010;79:437–442 20418035 10.1016/j.ejrad.2010.03.026

[7] Faure JM Mousty E Bigorre M . Prenatal ultrasound diagnosis of cleft palate without cleft lip, the new ultrasound semiology. Prenat Diagn 2020;40:1447–1458 32673416 10.1002/pd.5794

[8] Sander FH Jørgensen DS Jakobsen LP . Prenatal detection of orofacial clefts in Denmark from 2009 to 2018. Ultrasound Obstetr Gynecol 2023;63:507–513 10.1002/uog.27488 37724632

[9] Yan S Fu F Li R . Exome sequencing improves genetic diagnosis of congenital orofacial clefts. Front Genet 2023;14:1252823 37745857 10.3389/fgene.2023.1252823 PMC10512413

[10] ERN CRANIO. Accessed December 23, 2024. http://www.ern-cranio.eu/

[11] Wiechers C Kagan KO . Fetal markers for the detection of infants with craniofacial malformation. Semin Fetal Neonatal Med 2021;26:101291 34593337 10.1016/j.siny.2021.101291

[12] Sosiaali- ja terveysministeriö. Accessed December 23, 2024. http://stm.fi/seulonnat/sikioseulonnat

[13] HSE. In National Women & Infants Health Programme. Accessed December 23, 2024. http://www.hse.ie/eng/about/who/acute-hospitals-division/woman-infants/clinical-guidelines/quick-summary-document-fetal-anatomy-ultrasound-2023-.pdf

[14] Ministerie van Volksgezondheid, Welzijn en Sport. Prenatale en Neonatale Screeningen. Accessed December 23, 2024. http://www.pns.nl/prenatale-screeningen/

[15] Ministerstwa Zdrowia i Narodowego Funduszu Zdrowia. Pacjent. Accessed December 23, 2024. http://pacjent.gov.pl/program-profilaktyczny/program-badan-prenatalnych

[16] 1177. *Fosterdiagnostik*. Accessed December 23, 2024. http://www.1177.se/Stockholm/barn--gravid/graviditet/undersokningar-under-graviditeten/fosterdiagnostik/

[17] Aldridge N Pandya P Rankin J . Detection rates of a national fetal anomaly screening programme: a national cohort study. BJOG Int J Obstetr Gynaecol 2022;130:51–58 10.1111/1471-0528.17287 36054171

[18] Guichoud Y Ezzi OE De Buys Roessingh A . Cleft lip and palate antenatal diagnosis: a swiss university center performance analysis. Diagnostics 2023;13:2479 37568842 10.3390/diagnostics13152479 PMC10416856

[19] Nguyen JQ Calabrese CE Heaphy KJ . Can Robin sequence be predicted from prenatal ultrasonography? J Oral Maxillofac Surg 2019;78:612–618 31758942 10.1016/j.joms.2019.10.015

[20] Rotten D Levaillant J Martinez H . The fetal mandible: a 2D and 3D sonographic approach to the diagnosis of retrognathia and micrognathia. Ultrasound Obstetr Gynecol 2002;19:122–130 10.1046/j.0960-7692.2001.00622.x 11876802

[21] De Jong-Pleij EAP Ribbert LSM Manten GTR . Maxilla–nasion–mandible angle: a new method to assess profile anomalies in pregnancy. Ultrasound Obstetr Gynecol 2010;37:562–569 10.1002/uog.7768 20922777

[22] Baeza-Pagador A Tejero-Martínez A Salom-Alonso L . Diagnostic methods for the prenatal detection of cleft lip and palate: a systematic review. J Clin Med 2024;13:2090 38610855 10.3390/jcm13072090 PMC11012824

[23] Dabadie A Quarello E Degardin N . Added value of MRI for the prenatal diagnosis of isolated orofacial clefts and comparison with ultrasound. Diagn Intervent Imaging 2016;97:915–921 10.1016/j.diii.2015.11.015 26969118

[24] Van Der Hoek-Snieders HEM Van Den Heuvel AJML Van Os-Medendorp H . Diagnostic accuracy of fetal MRI to detect cleft palate: a meta-analysis. Eur J Pediatr 2019;179:29–38 31797081 10.1007/s00431-019-03500-x PMC6942582

[25] Yan X Xing G Wang X . Diagnostic value and application of prenatal MRI and ultrasound in fetal cleft lip and palate. Contrast Media Mol Imaging 2022;2022:9410161 35655725 10.1155/2022/9410161 PMC9132648

[26] Mink van der Molen AB Van Breugel JMM Janssen NG . Clinical practice guidelines on the treatment of patients with cleft lip, alveolus, and palate: an executive summary J Clin Med 2021;10:4813 34768332 10.3390/jcm10214813 PMC8584510

[27] By the Working Group on Writing a European Guideline on Robin Sequence . European Guideline Robin Sequence An Initiative From the European Reference Network for Rare Craniofacial Anomalies and Ear, Nose and Throat Disorders (ERN-CRANIO). J Craniofac Surg 2023;35:279–361 37811988 10.1097/SCS.0000000000009701

[28] FOD Volksgezondheid. Vrijwillige zwangerschapsonderbreking. 2023. Accessed December 20, 2024. http://www.health.belgium.be/nl/gezondheid/zorg-voor-jezelf/levensbegin-en-einde/vrijwillige-zwangerschapsonderbreking

[29] Ministry Of Social Affairs And Health. 2023. Accessed December 20, 2024. http://stm.fi/en/termination-of-pregnancy/

[30] HSE. 2022. Accessed December 20, 2024. http://www2.hse.ie/conditions/abortion/how-to-get/when/

[31] SNS 24. 2024. Accessed December 20, 2024. http://www.sns24.gov.pt/tema/saude-da-mulher/interrupcao-voluntaria-da-gravidez/

[32] Pravno-Informacijski Sistem Republike Slovenije. Accessed December 20, 2024. http://pisrs.si/pregledPredpisa?id=ZAKO408

[33] Maarse W Boonacker CWB Lapid O . Professional opinion on oral cleft during pregnancy: a comparison between Israel and the Netherlands. Prenat Diagn 2015;35:544–548 25641702 10.1002/pd.4570

[34] Ahmed S Hewison J Green JM . Decisions about testing and termination of pregnancy for different fetal conditions: a qualitative study of European White and Pakistani Mothers of Affected Children. J Genet Couns 2008;17:560–572 18841453 10.1007/s10897-008-9176-x

[35] Hawkins A Stenzel A Taylor J . Variables influencing pregnancy termination following prenatal diagnosis of fetal chromosome abnormalities. J Genet Couns 2012;22:238–248 23001505 10.1007/s10897-012-9539-1

[36] Davalbhakta A Hall PN . The impact of antenatal diagnosis on the effectiveness and timing of counselling for cleft lip and palate. Br J Plast Surg 2000;53:298–301 10876253 10.1054/bjps.2000.3330

[37] Rey-Bellet C Hohlfeld J . Prenatal diagnosis of facial clefts: evaluation of a specialised counselling. Schweiz Med Wochenschr 2004;134:640–644 10.4414/smw.2004.10547 15609207

[38] Han HH Choi EJ Kim JM . The importance of multidisciplinary management during prenatal care for cleft lip and palate. Arch Plast Surg 2016;43:153–159 27019808 10.5999/aps.2016.43.2.153 PMC4807170

