# Supplementary material for: Organization of Prenatal Care in Orofacial Clefts and Suspected Robin Sequence: A European Survey
Source: J Craniofac Surg. 2025 Mar 27;36(8):3054–9. doi: 10.1097/SCS.0000000000011312 (PMC12537042; doi:10.1097/SCS.0000000000011312)
Supplement: SUPPLEMENTARY MATERIAL [file scs-36-03054-s001.pdf]

Supplemental Digital Content 1 – Survey

# European Survey on Prenatal Care in Orofacial Clefts & Micro-/Retrognathia

**Welcome to the survey!**

As part of the ERN CRANIO initiative, we are exploring how prenatal care for orofacial clefts and/or retro-/micrognathia is organized across European centers. To better understand and address any gaps in care, we have developed this survey to gather your expert insights. We appreciate your valuable contribution.

*Thank you for participating!*

* Vereist

## Informed consent

Your consent is required for the use of this pseudonymized data. All information collected through this questionnaire will be used for the above-mentioned research and possibly for workgroup discussions within ERN. The questionnaire is pseudonymous, and the data will be collected and stored in a pseudonymized manner. The collected data will be analyzed within 6 months and then stored for 10 years.

**By checking the box below, you consent to the collection, use, storage and publication of your data as provided in this questionnaire (and no other sources), confirm that you are completing the questionnaire voluntarily, and declare that you are over 18 years of age.** *

◯ I agree to the collection, use, storage and publication of my data in accordance with the above criteria.

## General

### What is your primary specialty? *

Please select all that apply and please specify your answer in the text box if you selected 'other'

- [ ] Genetics
- [ ] Gynecology/obstetrics
- [ ] Oral and maxillofacial surgery
- [ ] Pediatrics
- [ ] Plastic and reconstructive surgery
- [ ] Radiology
- [ ] Andere

### What is your function? *

Please specify your answer in the text box if you selected 'other'

- ◯ Medical specialist / consultant
- ◯ Nurse practitioner
- ◯ Midwife
- ◯ Andere

**What is the name of your center?** *

- ◯ Amsterdam University Medical Centre
- ◯ Children's Health Ireland
- ◯ Erasmus Medical Centre
- ◯ Haukeland University Hospital
- ◯ Helsinki University Hospital
- ◯ Hôpital Necker - Enfants malades
- ◯ Hospital de Sant Joan de Déu
- ◯ Hospital de Santa Maria
- ◯ Karolinska University Hospital
- ◯ Prof. dr. S. Popowski Regional Specialised Children's Hospital
- ◯ Radboud Medical Centre
- ◯ Riga Stradins University
- ◯ Sahlgrenska University Hospital
- ◯ San Bortolo Hospital
- ◯ San Gerardo Hospital
- ◯ Smile House - San Paolo Hospital
- ◯ Strasbourg University Hospital
- ◯ Tübingen University Hospital
- ◯ University Clinic Salzburg
- ◯ University Hospital 12 de Octubre
- ◯ University Hospital Leuven
- ◯ University Medical Centre Ljubljana
- ◯ University Medical Centre Utrecht
- ◯ University of Pécs Clinical Centre
- ◯ Uppsala University Hospital
- ◯ Vall d'Hebron University Hospital
- ◯ Andere

**How would you categorize your center?** *

Please specify your answer in the text box if you selected 'other'

- ◯ General hospital
- ◯ University hospital / teaching hospital
- ◯ Andere

## Prenatal screening

**Is there a government-regulated prenatal screening program in your country (within a legal framework)?** *

- ◯ No
- ◯ Yes
- ◯ I don't know

**Please specify when this prenatal screening program was introduced in your country and add a link to the program here:**

**How is reimbursement for prenatal screening (i.e., imaging/genetic testing) handled in your country?** *

Please specify your answer in the text box if you selected 'other'

- ◯ Entirely covered by social health insurance
- ◯ Partially covered by social health insurance (e.g., only ultrasound)
- ◯ Only covered by private or additional health insurance, or out-of-pocket payment
- ◯ State
- ◯ I don't know
- ◯ Andere

**Please explain the reimbursement here:** *

**Is prenatal screening centralized or assigned to dedicated clinics?** *

- ◯ Centralized
- ◯ Dedicated clinics
- ◯ Both
- ◯ I don't know

**If dedicated clinics, please specify here:**

**Does your center have a protocol or care path for prenatal screening?** *

- ◯ No
- ◯ Yes
- ◯ I don't know

**Please share the link to the website with a prenatal screening protocol here:**

**What is the incidence of prenatally detected orofacial clefts in your center?** *

- ◯ Rarely (1-2 cases per year)
- ◯ Occasionally (3-10 cases per year)
- ◯ Frequently (11-20 cases per year)
- ◯ Very frequently (21+ cases per year)
- ◯ I don't know

**What is the incidence of prenatally detected micro-/retrognathia in your center?** *

- ◯ Rarely (1-2 cases per year)
- ◯ Occasionally (3-10 cases per year)
- ◯ Frequently (11-20 cases per year)
- ◯ Very frequently (21+ cases per year)
- ◯ I don't know

## Prenatal imaging

**Which type(s) of imaging does the prenatal screening program include in your center?** *

Please select all that apply and please specify your answer in the text box if you selected 'other'.

- ☐ 2D Ultrasound
- ☐ 3D Ultrasound (*three-dimensional view of the fetus; visualization of fetal anatomy is much clearer*)
- ☐ 4D Ultrasound (*3D in motion; visualization of movements of the fetus*)
- ☐ Fetal CT
- ☐ Fetal MRI
- ☐ None
- ☐ Andere

**What steps are taken or what changes occur in the prenatal screening program when an orofacial cleft or micro-/retrognathia is detected during a routine ultrasound?** *

Please select all that apply and please specify your answer in the text box if you selected 'other'

- ☐ Additional 3D ultrasound
- ☐ Additional 4D ultrasound
- ☐ Additional detailed, diagnostic ultrasound (2D)
- ☐ Fetal MRI
- ☐ Fetal CT
- ☐ Andere

**At what gestational age(s) is a detailed, diagnostic ultrasound performed (in the prenatal screening program)?**

Please write your answer in weeks

**Who performs the ultrasound in your center?** *

Please select all that apply and please specify your answer in the text box if you selected 'other'

- [ ] Gynecologist
- [ ] Midwife
- [ ] Radiologist
- [ ] Sonographer
- [ ] Andere

**Which items are assessed during the routine ultrasound?**

Please select all that apply and please specify your answer in the text box if you selected 'other'

- [ ] Number of fetuses
- [ ] Viability
- [ ] Growth of the fetus(es)
- [ ] Placenta morphology and localization
- [ ] (Structural) anatomy of the fetus(es)
- [ ] Amniotic fluid
- [ ] I don't know
- [ ] Andere

**Do you use a special technique to assess the palate during an ultrasound?** *

If yes, please describe the technique here:

**What technique is used to assess the growth of the mandible in comparison to the maxilla during an ultrasound?** *

*E.g., maxilla–nasion–mandible (MNM) angle.*

Please write your answer here:

**Are there any indications for which an additional fetal MRI is performed in your center?** *

- ( ) No
- ( ) Yes

**What are the indications for an additional fetal MRI?**

Please specify here:

**What is screened for on an MRI?**

Please write your answer here:

**At what gestational age(s) is an additional fetal MRI performed?**

Please write your answer in weeks

**How many cases with an orofacial cleft were detected after birth, but were not seen prenatally on an US or MRI (including isolated cleft palate cases) (estimated % per year)?**

If known, please write your answer here and describe the type of orofacial cleft that was (frequently) not seen:

**How many cases with micro-/retrognathia were detected after birth, but were not seen prenatally on an US or MRI (estimated % per year)?**

If known, please write your answer here:

## Prenatal genetics

**Is prenatal genetic testing available in your center?** *

- ◯ No
- ◯ Yes

**What types of prenatal genetic testing are available in your center?** *

Please select all that apply and please specify your answer in the text box if you selected 'other'

- ☐ Array-based copy number variation (CNV) analysis
- ☐ Orofacial cleft / RS gene panel (trio-analysis)
- ☐ Whole Genome Sequencing (trio-WGS)
- ☐ Whole Exome Sequencing (trio-WES)
- ☐ I don't know
- ☐ Andere

**In how many cases do you recommend prenatal genetic testing for pregnancies diagnosed with orofacial clefts?** *

- ◯ Rarely (less than 10% of cases)
- ◯ Occasionally (10-30% of cases)
- ◯ Frequently (31-50% of cases)
- ◯ Very frequently (51-70% of cases)
- ◯ Almost always (71-100% of cases)
- ◯ I don't know

**In how many cases do you recommend prenatal genetic testing for pregnancies diagnosed with micro-/retrognathia?** *

- Rarely (less than 10% of cases)
- Occasionally (10-30% of cases)
- Frequently (31-50% of cases)
- Very frequently (51-70% of cases)
- Almost always (71-100% of cases)
- I don't know

**What factors influence your decision to recommend prenatal genetic testing in cases of orofacial clefts?** *

Please select all that apply and please specify your answer in the text box if you selected 'other'

- Severity of the cleft
- Presence of additional anomalies
- Family history
- Maternal age
- Findings on diagnostic imaging
- Parental request
- Not applicable
- Andere

**What factors influence your decision to recommend prenatal genetic testing in cases of micro-/retrognathia?** *

Please select all that apply and please specify your answer in the text box if you selected 'other'

- Presence of additional anomalies
- Family history
- Maternal age
- Findings on diagnostic imaging
- Parental request
- Not applicable
- Andere

**Who primarily conducts the counseling related to prenatal genetic testing for orofacial clefts or micro-/retrognathia in your center?** *

Please select all that apply and please specify your answer in the text box if you selected 'other'

- ☐ Clinical geneticist
- ☐ Gynecologist
- ☐ Pediatrician
- ☐ Plastic surgeon
- ☐ Midwife
- ☐ Nurse practitioner
- ☐ Not applicable
- ☐ Andere

**In how many cases with an orofacial cleft do parents agree to prenatal genetic testing (estimation)?** *

- ○ Rarely (less than 10% of cases)
- ○ Occasionally (10-30% of cases)
- ○ Frequently (31-50% of cases)
- ○ Very frequently (51-70% of cases)
- ○ Almost always (71-100% of cases)
- ○ I don't know

**In how many cases with micro-/retrognathia do parents agree to prenatal genetic testing (estimation)?** *

- ○ Rarely (less than 10% of cases)
- ○ Occasionally (10-30% of cases)
- ○ Frequently (31-50% of cases)
- ○ Very frequently (51-70% of cases)
- ○ Almost always (71-100% of cases)
- ○ I don't know

**What follow-up actions are typically taken if prenatal genetic testing indicates a genetic diagnosis associated with the orofacial cleft or micro-/retrognathia?** *

Please select all that apply and please specify your answer in the text box if you selected 'other'

- [ ] Referral to a geneticist
- [ ] Additional counseling sessions
- [ ] Detailed anomaly ultrasound or MRI
- [ ] Planning for specialized postnatal care, if applicable
- [ ] Discussion of termination options
- [ ] I don't know
- [ ] Andere

**In your experience, how often do prenatal genetic tests reveal additional information that changes the management of pregnancies diagnosed with orofacial clefts?** *

- ○ Always
- ○ Sometimes
- ○ Never

**In your experience, how often do prenatal genetic tests reveal additional information that changes the management of pregnancies diagnosed with micro-/retrognathia?** *

- ○ Always
- ○ Sometimes
- ○ Never

**Are there any legal requirements or guidelines in your country regarding genetic testing for prenatal diagnoses of orofacial clefts or micro-/retrognathia?** *

- ○ No
- ○ Yes
- ○ I don't know

**Please share a link to the website of the protocol/guideline here:**

[ ]

## Counseling

**Who is primarily responsible for providing counseling to parents following a prenatal diagnosis of orofacial cleft or micro-/retrognathia in your center?** *

Please specify your answer in the text box if you selected 'other'

- ◯ Clinical geneticist
- ◯ Gynecologist
- ◯ Midwife
- ◯ Pediatrician
- ◯ Plastic surgeon
- ◯ Psychologist
- ◯ Social worker
- ◯ Orofacial multidisciplinary cleft team
- ◯ I don't know
- ◯ Andere

**Who are involved in the multidisciplinary cleft team?** *

Please write your answer here:

**Is there a standardized counseling protocol in place for cases of prenatal orofacial clefts or micro-/retrognathia in your center?** *

- ◯ No
- ◯ Yes
- ◯ I don't know

**How many counseling sessions are usually offered to parents after a diagnosis of orofacial cleft or micro-/retrognathia?** *

- ◯ One
- ◯ Two to three
- ◯ Four to five
- ◯ More than five
- ◯ As needed basis
- ◯ I don't know

**Is there a follow-up procedure to ensure parents have understood the information provided during counseling sessions?** *

- ◯ Always
- ◯ Sometimes
- ◯ Never
- ◯ I don't know

**Do you feel there is adequate interdisciplinary support when counseling parents about orofacial clefts or micro-/retrognathia?** *

- ◯ Strongly agree
- ◯ Agree
- ◯ Neutral
- ◯ Disagree
- ◯ Strongly disagree

## Termination of pregnancy (TOP)

**Is it legal in your country to terminate the pregnancy when an orofacial cleft has been diagnosed and parents wish to terminate?** *

- ◯ No
- ◯ Yes
- ◯ I don't know

**Is it legal in your country to terminate the pregnancy when micro-/retrognathia has been diagnosed and parents wish to terminate?** *

- ◯ No
- ◯ Yes
- ◯ I don't known

**Until what gestational age is termination of pregnancy for these indications allowed?**

The answer is in <u>weeks</u>

- ◯ 1
- ◯ 2
- ◯ 3
- ◯ 4
- ◯ 5
- ◯ 6
- ◯ 7
- ◯ 8
- ◯ 9
- ◯ 10
- ◯ 11
- ◯ 12
- ◯ 13
- ◯ 14
- ◯ 15
- ◯ 16
- ◯ 17
- ◯ 18
- ◯ 19
- ◯ 20
- ◯ 21
- ◯ 22
- ◯ 23
- ◯ 24
- ◯ 25
- ◯ 26
- ◯ 27
- ◯ 28
- ◯ 29
- ◯ 30

**Where is a medical abortion performed in your country (for example for an orofacial cleft)?** *

Please specify your answer in the text box if you selected 'other'

- ◯ Own clinic
- ◯ Abortion clinic
- ◯ Own preference
- ◯ Not applicable
- ◯ Andere

**How often do parents choose to terminate a pregnancy following a diagnosis of orofacial cleft in your center?** *

- ◯ Rarely (less than 10% of cases)
- ◯ Occasionally (10-30% of cases)
- ◯ Frequently (31-50% of cases)
- ◯ Very frequently (51-70% of cases)
- ◯ Almost always (71-100% of cases)
- ◯ I don't know

**How often do parents choose to terminate a pregnancy following a diagnosis of micro-/retrognathia in your center?** *

- ◯ Rarely (less than 10% of cases)
- ◯ Occasionally (10-30% of cases)
- ◯ Frequently (31-50% of cases)
- ◯ Very frequently (51-70% of cases)
- ◯ Almost always (71-100% of cases)
- ◯ I don't know

**Is there a trend in the number of termination of pregnancies (TOPs) performed because of an orofacial cleft or micro-/retrognathia in your center (i.e., objectively based on tracked data)?** *

◯ No

◯ Yes, downward

◯ Yes, upward

◯ I don't know

**Is any published or accessible data available on TOPs from your center, government or from a national (cleft) registry?** *

◯ No

◯ Yes

◯ I don't know

**Please specify where the national statistics can be found:**

[ ]

## Final questions

**Are there currently ongoing trials in your country aimed at (improving) prenatal diagnostics and/or counseling for orofacial clefts or micro-/retrognathia?** *

- ◯ No
- ◯ Yes
- ◯ I don't know

**Do you have any additional remarks?**

Please write your answer here:

Deze inhoud is niet door Microsoft gemaakt noch goedgekeurd. De gegevens die u verzendt, zal worden gestuurd naar de eigenaar van het formulier.

Microsoft Forms
