# Supplementary material for: Organization of Prenatal Care in Orofacial Clefts and Suspected Robin Sequence: A European Survey
Source: J Craniofac Surg. 2025 Mar 27;36(8):3054–9. doi: 10.1097/SCS.0000000000011312 (PMC12537042; doi:10.1097/SCS.0000000000011312)
Supplement: SUPPLEMENTARY MATERIAL [file scs-36-03054-s002.pdf]

Supplemental Digital Content 2 – Tables

SUPPLEMENTAL TABLE 1. Overview of respondents' characteristics

Country	Center	Respondents	Profession of respondent(s)	Prenatally detected orofacial cleft (cases per year)	Prenatally detected micro-/retrognathia (cases per year)
**Austria**	University Clinic Salzburg	1	Oral and maxillofacial surgeon	3-10	1-2
**Belgium**	University Hospital Leuven	1	Gynecologist/obstetrician	11-20	3-10
**Finland**	Helsinki University Hospital	1	Nurse specialist	11-20	1-2
**France**	Strasbourg University Hospital	1	Gynecologist/obstetrician	11-20	3-10
	Hôpital Necker – Enfants malades	1	Oral and maxillofacial surgeon	21+	21+
**Germany**	Tübingen University Hospital	1	Pediatrician	11-20	3-10
**Hungary**	University of Pécs Clinical Centre	1	Gynecologist/obstetrician	11-20	1-2
**Ireland**	Children's Health Ireland	1	Nurse specialist	11-20	1-2
	Rotunda Hospital, Dublin	1	Gynecologist	3-10	1-2
**Italy**	Smile House – San Paolo Hospital	1	Oral and maxillofacial surgeon	21+	3-10
	San Bortolo Hospital	1	Gynecologist/obstetrician	3-10	3-10
**Latvia**	Riga Cleft Lip and Palate Center	1	Gynecologist	3-10	3-10
**Netherlands**	Erasmus Medical Centre	1	Nurse specialist	21+	1-2

	Radboud Medical Centre	1	Clinical geneticist	Unknown	Unknown
	University Medical Centre Utrecht	3	Pediatrician<br>Plastic and reconstructive surgeon<br>Psychologist	21+	3-10
**Norway**	Haukeland University Hospital	1	Plastic and reconstructive surgeon	11-20	1-2
**Poland**	Medical University of Silesia	2	Gynecologist/obstetrician<br>Pediatrician	3-10 up to 11-20	1-2 up to 3-10
	Prof. dr. S. Popowski Regional Specialised Children's Hospital	1	Oral and maxillofacial surgeon	21+	11-20
**Portugal**	Hospital de Santa Maria	1	Plastic and reconstructive surgeon	21+	1-2
**Slovenia**	University Medical Centre Ljubljana	1	Oral and maxillofacial surgeon	3-10	1-2
**Spain**	Hospital de Sant Joan de Déu	2	Gynecologist/obstetrician<br>Pediatrician	11-20	1-2 up to 11-20
	Vall d'Hebron University Hospital	1	Gynecologist/obstetrician	11-20	1-2
	University Hospital 12 de Octubre	1	Gynecologist/obstetrician	3-10	3-10
**Sweden**	Uppsala University Hospital	1	Plastic and reconstructive surgeon	11-20	Unknown
	Sahlgrenska University Hospital	1	Clinical geneticist	Unknown	Unknown
	Karolinska University Hospital	1	Plastic and reconstructive surgeon	3-10	1-2
**United Kingdom**	Alder Hey Children's Hospital	1	Nurse specialist	11-20	1-2

SUPPLEMENTAL TABLE 2. Overview of prenatal screening programs

**Prenatal imaging and genetic testing**	**n (%)**
Local protocol for prenatal care	
Yes	24 (77%)
No	6 (19%)
I don't know	1 (3%)
Types of imaging included in standard diagnostics	
2D Ultrasound	20 (65%)
3D Ultrasound	26 (84%)
4D Ultrasound	13 (42%)
Fetal CT	3 (10%)
Fetal MRI	13 (42%)
Other	5 (16%)
None	1 (3%)
Availability of genetic testing in center	
Yes	28 (90%)
No	3 (10%)
National guideline for prenatal genetic testing	
Yes	3 (10%)
No	25 (81%)
I don't know	3 (10%)
Types of prenatal genetic testing	
Array-based CNV analysis	18 (58%)
Orofacial cleft / RS gene panel (trio-analysis)	10 (32%)
Whole Genome Sequencing (trio-analysis)	9 (29%)
Whole Exome Sequencing (trio-analysis)	16 (52%)
Other	5 (16%)
I don't know	6 (19%)
Local protocol for prenatal counselling	
Yes	15 (48%)
No	12 (39%)
I don't know	4 (13%)

Abbreviations: CT = Computed tomography, MRI Magnetic resonance imaging, RS = Robin sequence

SUPPLEMENTAL TABLE 3. Overview of prenatal imaging (techniques) and the number of prenatally missed cases (per center)

	Country	Performer US	Technique palate assessment (US)	Technique mandible assessment (US)	Fetal MRI	Prenatally missed orofacial cleft	Prenatally missed micro-/retrognathia
**1**	Norway	Gynecologist Midwife	N/A	N/A	Yes	100% for isolated cleft palate, 5-10% for cleft lip	100%
**2**	Slovenia	Gynecologist	Unknown	Unknown	No	100% for isolated cleft palate	100%
**3**	Finland	Gynecologist Midwife	N/A	Unknown	No	100% for isolated cleft palate, 5-10% for cleft lip	95%
**4**	Ireland	Gynecologist Midwife Radiologist Sonographer	Unknown	Unknown	No	95% for cleft lip, 5% for isolated cleft palate	95%
**5**	Latvia	Gynecologist	Face anatomy, lips and palate according to ISUOG first and second trimester anatomy assessment guidelines	MNM	Yes	>90% for isolated cleft palate, 5% for cleft lip or cleft lip and palate,	10%
**6**	Belgium	Gynecologist	Direct 2D, 3D rendering	MNM, IFA	Yes	>90% for isolated cleft palate, <5% for cleft lip	Unknown
**7**	Italy	Gynecologist	N/A	MNM	No	80% for isolated cleft palate	30%
**8**	Sweden	Sonographer	Unknown	Unknown	Yes	70%	99%
**9**	Austria	Gynecologist	N/A	MNM	No	70%	90%

**10**	United Kingdom	Sonographer	Unknown	Unknown	Yes	50%	60%
**11**	Netherlands	Gynecologist Midwife Sonographer	Unknown	MNM	Yes	<50% for isolated cleft palate, 0% for cleft lip	Unknown
**12**	Hungary	Gynecologist Sonographer	N/A	MNM	Yes	30-40%	60-70%
**13**	Netherlands	Gynecologist Sonographer	Equal sign	IFA	No	30% for isolated cleft palate	3%
**14**	Germany	Gynecologist	Axial transverse ultrasound view, equal sign	MNM, IFA, FPL, PFSR, FS	No	20%	10%
**15**	Sweden	Gynecologist Midwife Sonographer	N/A	Length measurement maxilla and mandible	No	10-20%	1-2%
**16**	Spain	Gynecologist Sonographer	Search mouth bell, Batman sign, palatine bone visualization	MNM, IFA	Yes	10% (mainly cleft palate)	10%
**17**	Poland	Radiologist	N/A	Profile/sagittal view imaging, jaw-to-face ratio, jaw index, mandibular length, IFA	No	7%	5%
**18**	Italy	Gynecologist	3D US	MNM	Yes	1%	1%
**19**	France	Gynecologist Radiologist	N/A	Unknown	Yes	<1%	<5%
**20**	Spain	Gynecologist	N/A	N/A	Yes	<1%	<1%

**21**	France	Gynecologist Midwife	Horizontal place that check the posterior wall of palate between the 2 sphenoid bone; sagittal place during deglutition	N/A	Yes	<1%	Unknown
**22**	Spain	Gynecologist	N/A	MNM, jaw index, mandible length	Yes	0%	Unknown
**23**	Ireland	Sonographer	N/A	N/A	No	Unknown	A few
**24**	Sweden	Gynecologist Midwife	Unknown	Unknown	Yes	Unknown	Unknown
**25**	Portugal	Gynecologist	Unknown	Unknown	No	Unknown	Unknown
**26**	Poland	Fetal cardiologist Gynecologist Pediatrician	Sagittal frontal view (2D) + 3D, CD used during fetal swallowing	MNM	Yes	Unknown	Unknown
**27**	Netherlands	Sonographer	Unknown	Unknown	Yes	Unknown	Unknown

Abbreviations: CD = Color Doppler, FPL = Fetal profile line, FS = Frontal space, IFA = Inferior facial angle, MNM = Maxilla-nasion-mandible ratio, PFSR = Prefrontal space ratio
